# Case report: quantification of methadone-induced respiratory depression using toxicokinetic/toxicodynamic relationships

**DOI:** 10.1186/cc5150

**Published:** 2007-01-15

**Authors:** Bruno Mégarbane, Xavier Declèves, Vanessa Bloch, Christophe Bardin, François Chast, Frédéric J Baud

**Affiliations:** 1 INSERM U705, CNRS, UMR 7157; Université Paris 7; Assistance Publique – Hôpitaux de Paris, Hôpital Lariboisière, Réanimation Médicale et Toxicologique, 2 Rue Ambroise Paré, 75010, Paris, France; 2 INSERM U705, CNRS, UMR 7157; Université Paris 7; Assistance Publique – Hôpitaux de Paris, Hôpital Fernand Widal, 200 Rue du Faubourg Saint Denis, 75018, Paris, France; 3 Assistance Publique – Hôpitaux de Paris, Hôpital Hôtel-Dieu, Laboratoire de Toxicologie, 1 Place Notre-Dame 75004, Paris, France

## Abstract

**Introduction:**

Methadone, the most widely delivered maintenance therapy for heroin addicts, may be responsible for life-threatening poisonings with respiratory depression. The toxicokinetics and the toxicokinetic/toxicodynamic (TK/TD) relationships of methadone enantiomers have been poorly investigated in acute poisonings. The aim of this study was to describe the relationships between methadone-related respiratory effects and their corresponding concentrations.

**Methods:**

We report a 44-year-old methadone-maintained patient who ingested a 240-mg dose of methadone. He was found comatose with pinpoint pupils and respiratory depression. He was successfully treated with intravenous naloxone infusion over the course of 31 hours at a rate adapted to maintain normal consciousness and respiratory rate. We performed a TK/TD analysis of the naloxone infusion rate needed to maintain his respiratory rate at more than 12 breaths per minute (as toxicodynamics parameter) versus plasma *R*,*S*- and *R*-methadone concentrations (as toxicokinetics parameter), determined using an enantioselective high-performance liquid chromatography assay.

**Results:**

Initial plasma *R*,*S*-methadone concentration was 1,204 ng/ml. Decrease in plasma *R*- and *S*-methadone concentrations was linear and demonstrated a first-order pharmacokinetics (maximal observed concentrations 566 and 637 ng/ml, half-lives 16.1 and 13.2 hours, respectively). TK/TD correlation between naloxone infusion rate and *R*,*S*- and *R-*methadone concentrations fitted well a sigmoidal *E*_*max *_model (concentration associated with a half-maximum effect [*EC*_50_] 334 and 173 ng/ml, Hill coefficient 10.0 and 7.8, respectively). In our chronically treated patient, *EC*_50 _values were in the range of previously reported values regarding methadone analgesic effects, suggesting that plasma methadone concentrations to prevent withdrawal are lower than those associated with methadone analgesic effects.

**Conclusion:**

After the ingestion of a toxic dose of a racemic mixture, plasma *R*- and *S*-enantiomer concentrations decreased in parallel. Despite large inter-individual variability in methadone toxicokinetics and toxicodynamics, TK/TD relationships would be helpful for providing quantitative data regarding the respiratory response to methadone in poisonings. However, further confirmatory TK/TD data are needed.

## Introduction

Methadone has been the most widely delivered maintenance therapy for heroin users for almost 50 years. Prescribed with doses adequate to the actual needs of individual addicts, methadone efficiently reduces heroin use, overdose mortality, as well as criminal activities [1-3]. The major pharmacological activity of methadone, a long-lasting opioid agonist, is related to its binding to the mu-opioid receptors. The available marketed methadone (6-dimethylamino-4,4-diphenyl-3-heptanone) is a racemic mixture of two stereoisomeric forms, the *R*- and *S*-enantiomers [4]. *R*-methadone is the main pharmacologically active isomer, believed to account for most if not all of the therapeutic effects of methadone maintenance treatment. However, although lacking strong opioid effects, *S*-methadone may play a significant role in the adverse responses to *R*,*S*-methadone [5-7].

Opioid toxicity produces a classic syndrome characterised by a depressed level of consciousness, bradypnea, and miosis. Methadone overdose results in life-threatening respiratory depression, which may be reversed using naloxone, a mu-receptor competitive antagonist [8]. Regardless of whether initial doses of naloxone restore adequate respiration and further therapy is needed, repeated boluses or continuous infusion of naloxone can be used [9,10]. In the setting of close monitoring of neurological and respiratory parameters, naloxone may obviate the need for tracheal intubation and mechanical ventilation.

To our knowledge, toxicokinetic/toxicodynamic (TK/TD) relationships in regard to respiratory depression have not been studied in methadone poisonings. Moreover, the relative kinetics of each methadone enantiomer after the ingestion of a toxic dose remains unclear. Here, we report a case of methadone poisoning with severe respiratory depression treated using naloxone. We performed a toxicokinetics study of both *S*- and *R*-methadone enantiomers and a TK/TD study to understand the relationships between the respiratory effects and the concentrations of the active *R*-enantiomer.

## Materials and methods

A 44-year-old man was found unconscious outdoors and admitted to the emergency department of a neighbouring hospital. On presentation, he was comatose (Glasgow Coma Score 3) with a blood pressure of 118/71 mm Hg, a heart rate of 117 beats per minute, a respiratory rate of 10 breaths per minute, and an SpO_2 _(percutaneous oxygen saturation) of 75%. His temperature was 36.5°C. Neurological examination showed hypotonic and hyporeflexic coma with pinpoint pupils. Pulmonary auscultation was normal. Arterial blood gas analysis while breathing on room air showed a pH of 7.30, a PaO_2 _(arterial oxygen pressure) of 56 mm Hg, PaCO_2 _(arterial carbon dioxide pressure) of 48 mm Hg, and a serum bicarbonate concentration of 26 mmol/l. Other routine laboratory tests were unremarkable. Plasma lactate concentration was 2.1 mmol/l. Significant improvement in consciousness level (Glasgow Coma Score 15) without mental confusion was obtained after an intravenous bolus injection of 0.3 mg of naloxone followed by a continuous 0.3-mg/hour infusion. The patient rapidly awoke and declared that he had intentionally ingested 240 mg (3.14 mg/kg) of methadone and 2 mg of flunitrazepam to relax. The delay between ingestion and admission to the hospital was estimated to be 2.7 hours. He had a history of heroin addiction with a maintenance treatment of 70 mg/day of methadone (confirmed by a prescription from his maintenance program centre) and occasional use of 2 mg of flunitrazepam. He suffered from depression, and a recently diagnosed hyperthyroidism was treated with neomercazole. He was transferred for monitoring to our intensive care unit (ICU). The attending physicians managed the patient according to the standard treatment guidelines currently used in our department. Naloxone infusion rate was progressively adapted, aiming to keep the patient calm with a normal mental status (Glasgow Coma Score 15) and a respiratory rate of more than 12 breaths per minute. Reduction in naloxone dose was systematically attempted, and when evidence of toxicity occurred, a return to the previous dose was made. Routine toxicological screening of blood and urine, including benzodiazepines, opiates, buprenorphine, cocaine, and tetrahydrocannabinol, was negative, except for methadone. This case outcome was favourable with an uneventful 48-hour ICU stay, except for an aspiration pneumonia treated with amoxicillin-clavulanic acid for six days. The duration of naloxone intravenous infusion was 31 hours with a cumulative dose of 9.1 mg.

*R*-methadone accounts for most, if not all, of the effect of methadone [5-7]. To this extent, we considered that TK/TD relationships in regard to methadone respiratory effects for *S*-enantiomer would probably just mirror those of *R*-enantiomer. Therefore, we performed a TK/TD analysis of the naloxone infusion rate needed to maintain the respiratory rate of more than 12 breaths per minute (as toxicodynamics parameter) versus plasma *R*,*S*- and *R*-methadone concentrations (as toxicokinetics parameter). Upon admission to the ICU, verbal informed consent was obtained from the completely awakened and non-confused patient. Venous blood samples were obtained at 2.7, 10.5, 14.5, 18.5, 20.5, 22.5, 26.5, and 30.5 hours after methadone ingestion. Plasma was separated and frozen until methadone concentration measurement. The rate of naloxone infusion was prospectively collected at each blood sampling time, and the nurses in charge were blinded to the results of the toxicological analyses. Both *R*- and *S*-methadone enantiomer concentrations were measured using a validated chiral high-performance liquid chromatography assay as previously described [11]. The plasma kinetics of each enantiomer were analysed using a noncompartmental method. Toxicokinetics parameters and TK/TD relationships were determined using a computerised curve-fitting program (WinNonlin Pro 3.0; Pharsight Corporation, Mountain View, CA, USA). The area under the curve from 0 to infinity (*AUC*_*inf*_) was calculated using the model-independent trapezoidal method.

## Results

**Figure 1:**
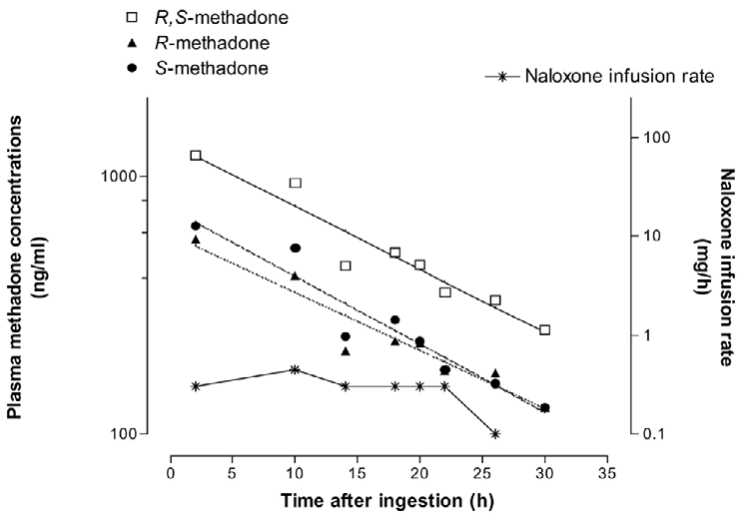
Plasma *R*,*S*-, *R*-, and *S*-methadone concentrations (left y-axis) and naloxone infusion rate (right y-axis) versus time after ingestion of a 3.14-mg/kg single oral dose of the racemic *R*,*S*-methadone.

**Figure 2:**
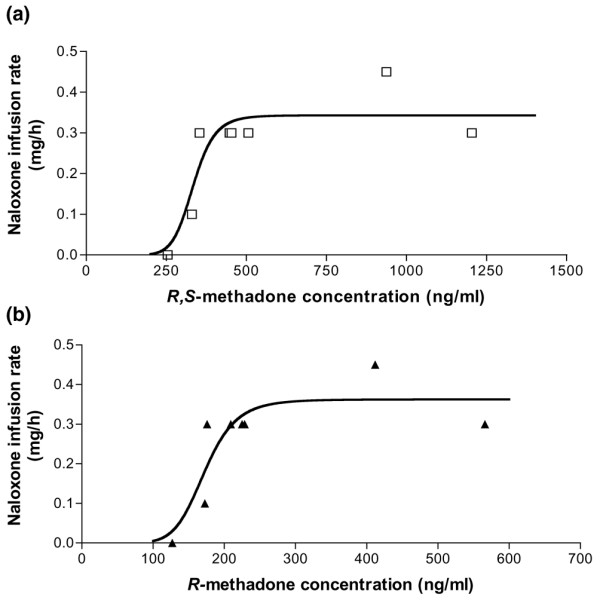
Toxicokinetic/toxicodynamic relationships between the naloxone infusion rate and the plasma concentrations of **(a) **the racemic *R*,*S*-methadone and **(b) **the *R*-methadone enantiomer.

The maximal observed plasma concentration of *R*,*S*-methadone was 1,204 ng/ml at 2.7 hours (Table 1). Decrease in plasma *R*,*S*-, *R*-, and *S*-methadone concentrations was of first-order pharmacokinetics (Figure 1). During the course of overdose after naloxone infusion, the respiratory rate was maintained at more than 12 breaths per minute (therapeutic objective). The Glasgow Coma Score was constantly at 15, and the haemodynamic parameters were within the normal range. TK/TD correlations between naloxone infusion rate (*E*) and *R*,*S*- and *R*-methadone concentrations (*C*) fitted well the sigmoidal *E*_*max *_model, *E *= *E*_*max*_*C*^*γ*^/[*EC*_50_^*γ *^+ *C*^*γ*^] + *E*_0_, where *E*_0 _is the baseline value of the measured rate of naloxone infusion, *E*_*max *_represents the maximum possible infusion rate, *EC*_50 _represents the concentration associated with the half-maximum infusion rate, and *γ *(or Hill coefficient) determines the steepness (slope) of the concentration-versus-response curve (Figure 2). Values of *EC*_50 _and *γ *are given in Table 2.

**Table 1:** Toxicokinetic parameters of the racemic *R*,*S*-methadone and both *R*- and *S*-methadone forms in a severely poisoned patient

	*T*_1/2_	Maximal observed concentration	*AUC*_*inf*_	*V*_*z*_/*F*	*Cl*_*t*_/*F*
	(h)	(ng/ml)	(ng-h/ml)	(l)	(l/h)

*R*-methadone	16.1	566	13,241	211	9.1
*S*-methadone	13.2	637	13,385	170	9.0
*R*,*S*-methadone	14.5	1,204	26,508	189	9.1

*AUC*_*inf*_, area under the curve from 0 to infinity; *Cl*_*t*_/*F*, total plasma clearance; *T*_1/2_, half-life; *V*_*z*_/*F*, distribution volume.

**Table 2:** Parameters of the TK/TD relationships model regarding the naloxone infusion rate versus *R*,*S*- and *R*-methadone concentrations

	*EC*_50_	*γ*
	(ng/ml)	

*R*-methadone	173	7.8
*R*,*S*-methadone	334	10.0

*γ*, Hill coefficient; *EC*_50_, concentration associated with a half-maximum effect; *E*_*max*_, maximum effect; TK/TD, toxicokinetic/toxicodynamic.

## Discussion

Methadone pharmacokinetics and pharmacodynamics are characterised by a high inter-individual variability, even in toxic conditions [7,12,13]. There is up to 17-fold inter-individual variation of blood methadone concentrations for a given dosage [7]. Because methadone is administered as a racemic mixture, our objective was to study the relationships between the respiratory effects and the racemic as well as the active *R*-enantiomer concentrations.

*R*-methadone is believed to account for most, if not all, of the therapeutic effects of methadone maintenance treatment [5-7,14]. *S*-methadone may be a clinically important determinant of adverse responses to racemic methadone and may significantly contribute to the adverse but not the therapeutic effects of racemic methadone during maintenance treatment for opioid dependence. In healthy volunteers, 7.5 mg of oral *S*-methadone did not significantly differ from the placebo response in respiratory and pupillary effects, whereas 7.5 mg of *R*-methadone and 15 mg of *R*,*S*-methadone induced dramatic and sustained respiratory depression and miosis [15]. In the same volunteers, *S*-methadone doses between 50 and 100 mg slightly depressed ventilation. In dependent patients, *S*-methadone administered at dosages of 650 to 1,000 mg/day induced subjective morphine-like effects, partially suppressed abstinence from morphine, and created a mild degree of physical dependence [16].

At therapeutic doses, plasma concentrations of *R*,*S*-methadone decrease in a bi-exponential manner, with an elimination half-life estimated at 22 ± 7 hours (range 14 to 40) in opioid users after 26 days of methadone [7]. Trough plasma concentrations of 400 ng/ml for *R*,*S*-methadone or 250 ng/ml for *R*-methadone are usual target values when measurement of methadone concentration is performed to investigate a non-complete clinical response [17]. In our overdosed patient, the maximal observed *R*,*S*-methadone concentration was approximately three times higher than the reported peak levels in patients treated with a daily therapeutic dosage of more than 80 mg [18].

The almost mono-exponential first-order decline in plasma *R*- and *S*-methadone concentrations suggests the absence of any saturation of the distribution and the elimination processes, even at supra-therapeutic concentrations. Methadone toxicokinetics was in close agreement with pharmacokinetics, with an observed half-life in the lower range of the reported pharmacokinetic half-lives [7]. Methadone is metabolised mainly into EDDP (2-ethylidene-1,5-dimethyl-3,3-diphenylpyrrolidine) through cytochrome P450 (CYP) 3A4 and 2B6 pathways, for which genetic polymorphisms and high inter-individual variability of activity were reported, resulting in large inter-individual variability of methadone half-lives. *In vitro *metabolism studies using human liver microsomes showed apparent affinity constants (K_m_) regarding CYP-mediated EDDP formation far above the maximal observed methadone concentration in this patient [19], suggesting that saturation of methadone metabolism did not occur in our patient. Moreover, no study of methadone dose-linearity was performed at such elevated concentrations. Thus, we may hypothesise that similar data (first-order kinetics) would have been observed with a larger data set of intoxicated patients presenting similar plasma methadone concentrations. Both enantiomer half-lives were rather short in comparison to previously reported values in eight patients undergoing methadone maintenance treatment (*R*-methadone 23 to 50 hours and *S*-methadone 16 to 37 hours) [20]. Thus, although no definitive explanation could be given, we may suggest that our patient was an extensive CYP 3A4 metaboliser, because no CYP 3A-inducing drugs were identified in his treatment. Moreover, as previously reported [7], the half-life of *R*-methadone was longer than that of *S*-methadone.

TK/TD relationships allow the descriptive and quantitative characterisation of the time course of the *in vivo *drug effect in relation to its corresponding drug concentrations within an individual [21]. There are several studies dealing with pharmacokinetic/pharmacodynamic relationships for opioids commonly used in anaesthesia or for the treatment of pain [22,23]. However, only a limited number of studies addressed the pharmacokinetic/pharmacodynamic relationships of methadone in healthy volunteers, patients treated for chronic pain, or maintenance patients [5,23-25]. To our knowledge, there is no case of human methadone poisoning with a TK/TD analysis of respiratory effects. In our patient, we found sigmoidal TK/TD relationships. The maximal naloxone infusion rate was associated with an *R*, *S*-methadone concentration ranging from 400 to 1,200 ng/ml, suggesting a saturation of the mu-opioid receptors at these concentrations. Similarly, a study in healthy volunteers showed a sigmoidal response with minimal hysteresis between the iris/pupil ratio and its corresponding plasma *R*-methadone concentration (*EC*_50 _2.3 ng/ml and *γ *9.0) [5]. In patients with chronic pain, pain relief was related to the *R*, *S-*methadone concentrations by a sigmoid *E*_*max *_model defined with an *EC*_50 _of 290 ng/ml and a steepness of 2.03 [25]. In our case, the high value of *γ *(10.0) clearly indicated that a small decrease in plasma concentrations near the *EC*_50 _was associated with a dramatic decrease in the rate of naloxone infusion. *R*-methadone exhibited a lower *γ *than *R*,*S*-methadone, suggesting that *S*-methadone enantiomer could demonstrate a positive allosteric cooperation with *R*-methadone activity. However, in the present case, the confidence intervals regarding the *γ *values were largely overlapping, precluding any definitive conclusion. *R*,*S-*methadone *EC*_50 _of 334 ng/ml was associated with significant respiratory effects in our patient. This concentration was less elevated than the target concentration of 400 ng/ml in patients treated with elevated doses, suggesting that our patient was probably equilibrated with lower methadone concentrations. However, a relationship if any between *EC*_50 _and a therapeutic drug monitoring tool, as the trough plasma *R*,*S*-methadone concentration need to be established.

Moreover, although we may hypothesise that the *EC*_50 _values regarding methadone respiratory effects in our patient are higher than values that could have been observed in opiate-naïve patients due to a pharmacodynamic tolerance, these *EC*_50 _values were in the same range as those obtained for pain relief in chronically treated patients [25]. Increase of EC_50 _could also be related in our patient to a pharmacogenetic variant of mu-receptor, as previously reported [26]. Thus, our data clearly support the tight safety index of methadone, previously illustrated by the occurrence of significant respiratory effects after intake at pharmacological dosages [27]. Consistently, during the first months of methadone maintenance, there is a continual alveolar hypoventilation due to the depression of both central and peripheral chemoreception [28]. Interestingly, alteration in ventilatory response to hypoxia persists after a prolonged treatment, although alveolar hypoventilation is abolished after the fully acquired tolerance within two months of the carbon dioxide-sensitive chemoreflex. However, this adaptation of ventilatory response to chronic use of methadone does not prevent the occurrence of alveolar hypoventilation in the case of acute methadone overdose.

Our toxicokinetics study has significant limitations. First, *AUC*_*inf *_could be slightly underestimated due to the absence of any sample before 2.7 hours post-ingestion and between 2.7 and 10.5 hours, as the mean maximal observed concentration has been previously reported between 2.5 and 4.4 hours post-ingestion [29]. The bioavailability of methadone is also unknown in such an overdose situation. In addition, the AUC versus time was calculated between 0 and infinity as if our patient ingested a single dose of methadone, regardless of a possible long-term maintenance therapy, which was not certain in the absence of previous therapeutic drug monitoring. Thus, the apparent volume of distribution (*V*_*z*_/*F*) and the total plasma clearance (*Cl*_*t*_/*F*) values calculated using *AUC*_*inf *_should be interpreted with caution. However, *Cl*_*t*_/*F *and *V*_*z*_/*F *values for both methadone enantiomers were comparable to those reported in patients undergoing methadone maintenance at therapeutic dosage [20], suggesting the absence of saturation of methadone pharmacokinetics in this case. The *V*_*z*_/*F *values of *R*- and *S*-methadone (211 versus 170 l, respectively) were lower than those reported in treated patients (470 versus 318 l, respectively [20]) but similar to those reported in healthy volunteers (106 versus 262 l, respectively [7]). We have no definitive explanations for these differences, due to the imprecision of the *AUC*_*inf *_calculation. However, methadone *V*_*z*_/*F *is influenced by the acid α-1-glycoprotein levels with regard to its transfer to and from the central to the peripheral compartments and by extension to the site of methadone pharmacological action [7]. The *Cl*_*t*_/*F *was similar for both methadone *R*- and *S*-enantiomers (9.0 versus 9.1 l/hour, respectively) and close to values reported in treated patients (10.7 versus 9.7 l/hour, respectively [20]). In contrast, in healthy adults, the pharmacologically active *R*-enantiomer has a lower *Cl*_*t*_/*F *when compared with *S*-methadone (4.0 versus 20.4 l/hour, respectively) [7].

We considered the naloxone infusion rate as a surrogate marker of methadone-induced respiratory depression. However, because the elimination rate of naloxone varies with a half-life of 45 to 90 minutes, a continuous infusion runs the risk of naloxone accumulation, complicating the pharmacodynamic interactions. Otherwise, such a toxicodynamics surrogate marker requires a close concordance between real effects on respiration and the titrated infusion rate of naloxone. Moreover, serum and urine specimens were not tested for flunitrazepam in our patient. Consistently, one unusual feature in this case is the rapidity with which the patient appears to have suffered unconsciousness. Given that the peak effects of methadone usually occur approximately four to six hours after ingestion [7] and that flunitrazepam is a short-acting agent with respect to methadone, we think the initial presentation may have been influenced by the co-ingestion of flunitrazepam. However, several studies suggest that methadone peak plasma concentrations and effects may occur much earlier [30,31], supporting (as in our patient) methadone-related steep concentration-effect relationships. Otherwise, we cannot rule out potential interactions between flunitrazepam and methadone that contributed to the respiratory depression. Both molecules are metabolised by the CYP 3A4, which may lead to clinically significant drug-drug interactions. However, the relatively short half-life of *R*, *S*-methadone does not support any inhibition of CYP 3A4-mediated methadone metabolism by flunitrazepam.

Sources of inter-individual variability of the response to methadone are numerous, including demographic and physiopathological characteristics of the methadone-maintained subjects [23,32]. Thus, analysis of the TK/TD relationships in one patient precludes any definitive conclusion regarding the various possible situations of exposures to methadone in other patients. In addition, because plasma is not the effect site of methadone and time is needed before the drug distributes into the central nervous system, another significant concern in our study is the absence of characterisation of the plasma-versus-biophase distribution. For some opioids, there may be a delay between the time course of the plasma concentrations and the time course of the effects [5,22,25]. Moreover, the rate of plasma protein binding was not determined in this patient. However, because methadone is a highly lipophilic drug, the rate of cerebral distribution of the pharmacologically active molecules should not be a limiting step in this study of TK/TD relationships at toxic concentrations. Here, we described TK/TD relationships during the methadone elimination phase.

## Conclusion

Plasma toxicokinetics of *R*- and *S*-methadone enantiomers were parallel. The study of TK/TD relationships appears to be helpful for quantifying the respiratory response to methadone during poisonings. Despite a possible pharmacodynamic tolerance in our patient, the *EC*_50 _values regarding the methadone respiratory effects were in the same range as those previously reported for the analgesic effects in chronically treated patients, thus suggesting a limited safety index for methadone. A better understanding of the toxicokinetics and toxicodynamics of methadone and its enantiomers will be possible as more data accumulate in this regard.

## Key messages

• Methadone may be responsible for life-threatening poisonings with respiratory depression.

• The pharmacokinetics of plasma *R*- and *S*-methadone enantiomers is of first order, with elimination half-lives of 16.1 and 13.2 hours, respectively.

• The TK/TD correlation between the naloxone infusion rate needed to maintain a respiratory rate of more than 12 breaths per minute and *R*,*S*- and *R-*methadone concentrations fits well a sigmoidal *E*_*max *_model (*EC*_50 _334 and 173 ng/ml, *γ *10.0 and 7.8, respectively).

• Despite large inter-individual variability, TK/TD relationships would be helpful for providing quantitative data on the respiratory response to methadone in poisonings.

• In the reported chronically treated patient, *EC*_50 _values are in the range of the previously described values for methadone analgesic effects.

## Abbreviations

*γ *= Hill coefficient; *AUC*_*inf *_= area under the curve from 0 to infinity; *Cl*_*t*_/*F *= total plasma clearance; CYP = cytochrome P450; *EC*_50 _= concentration associated with a half-maximum effect; EDDP = 2-ethylidene-1,5-dimethyl-3,3-diphenylpyrrolidine; *E*_*max *_= maximum effect; ICU = intensive care unit; TK/TD = toxicokinetic/toxicodynamic; *V*_*z*_/*F *= distribution volume.

## Competing interests

The authors declare that they have no competing interests.

## Authors' contributions

BM and FJB were in charge of the patient, coordinated the data analysis, and drafted the manuscript. XD and VB performed the TK analysis and the study of TK/TD relationships. CB and FC contributed to the measurements of methadone concentrations. All authors read and approved the final manuscript.

## References

[B1] Farrell M, Ward J, Mattick R, Hall W, Stimson GV, des Jarlais D, Gossop M, Strang J (1994). Methadone maintenance treatment in opiate dependence: a review. BMJ.

[B2] Mattick RP, Breen C, Kimber J, Davoli M (2003). Methadone maintenance therapy versus no opioid replacement therapy for opioid dependence. Cochrane Database Syst Rev.

[B3] Krantz MJ, Mehler PS (2004). Treating opioid dependence. Growing implications for primary care. Arch Intern Med.

[B4] Kristensen K, Christensen CB, Christrup LL (1995). The mu1, mu2, delta, kappa opioid receptor binding profiles of methadone stereoisomers and morphine. Life Sci.

[B5] Boulton DW, Arnaud P, DeVane CL (2001). Pharmacokinetics and pharmacodynamics of methadone enantiomers after a single oral dose of racemate. Clin Pharmacol Ther.

[B6] Scott CC, Robbins EB, Chen KK (1948). Pharmacologic comparison of the optical isomers of methadone. J Pharmacol Exp Ther.

[B7] Eap CB, Buclin T, Baumann P (2002). Interindividual variability of the clinical pharmacokinetics of methadone: implications for the treatment of opioid dependence. Clin Pharmacokinet.

[B8] Corkery JM, Schifano F, Ghodse AH, Oyefeso A (2004). The effects of methadone and its role in fatalities. Hum Psychopharmacol.

[B9] Zimmerman JL (2003). Poisonings and overdoses in the intensive care unit: general and specific management issues. Crit Care Med.

[B10] Goldfrank L, Weisman RS, Errick JK, Lo MW (1986). A dosing nomogram for continuous infusion intravenous naloxone. Ann Emerg Med.

[B11] Batista R, Badre-Sentenac S, Bardin C, Chast F (2004). [Plasma assay of methadone enantiomers with high performance liquid chromatography]. Ann Pharm Fr.

[B12] Foster DJ, Somogyi AA, White JM, Bochner F (2004). Population pharmacokinetics of (R)-, (S)- and rac-methadone in methadone maintenance patients. Br J Clin Pharmacol.

[B13] Hanna J, Foster DJ, Salter A, Somogyi AA, White JM, Bochner F (2005). Within- and between-subject variability in methadone pharmacokinetics and pharmacodynamics in methadone maintenance subjects. Br J Clin Pharmacol.

[B14] Mitchell TB, Dyer KR, Newcombe D, Salter A, Somogyi AA, Bochner F, White JM (2004). Subjective and physiological responses among racemic-methadone maintenance patients in relation to relative (S)- vs. (R)-methadone exposure. Br J Clin Pharmacol.

[B15] Olsen GD, Wendel HA, Livermore JD, Leger RM, Lynn RK, Gerber N (1977). Clinical effects and pharmacokinetics of racemic methadone and its optical isomers. Clin Pharmacol Ther.

[B16] Fraser HF, Isbell H (1962). Human pharmacology and addictiveness of certain dextroisomers of synthetic analgesics. Bull Narc.

[B17] Eap CB, Bourquin M, Martin J, Spagnoli J, Livoti S, Powell K, Baumann P, Deglon J (2000). Plasma concentrations of the enantiomers of methadone and therapeutic response in methadone maintenance treatment. Drug Alcohol Depend.

[B18] Crettol S, Deglon JJ, Besson J, Croquette-Krokkar M, Gothuey I, Hammig R, Monnat M, Huttemann H, Baumann P, Eap CB (2005). Methadone enantiomer plasma levels, CYP2B6, CYP2C19, and CYP2C9 genotypes, and response to treatment. Clin Pharmacol Ther.

[B19] Kharasch ED, Hoffer C, Whittington D, Sheffels P (2004). Role of hepatic and intestinal cytochrome P450 3A and 2B6 in the metabolism, disposition, and miotic effects of methadone. Clin Pharmacol Ther.

[B20] Benmebarek M, Devaud C, Gex-Fabry M, Powell Golay K, Brogli C, Baumann P, Gravier B, Eap CB (2004). Effects of grapefruit juice on the pharmacokinetics of the enantiomers of methadone. Clin Pharmacol Ther.

[B21] Baud FJ (1998). Pharmacokinetic-pharmacodynamic relationships. How are they useful in human toxicology?. Toxicol Lett.

[B22] Lotsch J (2005). Pharmacokinetic-pharmacodynamic modeling of opioids. J Pain Symptom Manage.

[B23] Garrido MJ, Troconiz IF (1999). Methadone: a review of its pharmacokinetic/pharmacodynamic properties. J Pharmacol Toxicol Methods.

[B24] Inturrisi CE, Portenoy RK, Max MB, Colburn WA, Foley KM (1990). Pharmacokinetic-pharmacodynamic relationships of methadone infusions in patients with cancer pain. Clin Pharmacol Ther.

[B25] Inturrisi CE, Colburn WA, Kaiko RF, Houde RW, Foley KM (1987). Pharmacokinetics and pharmacodynamics of methadone in patients with chronic pain. Clin Pharmacol Ther.

[B26] Lotsch J, Skarke C, Wieting J, Oertel BG, Schmidt H, Brockmoller J, Geisslinger G (2006). Modulation of the central nervous effects of levomethadone by genetic polymorphisms potentially affecting its metabolism, distribution, and drug action. Clin Pharmacol Ther.

[B27] Santiago TV, Goldblatt K, Winters K, Pugliese AC, Edelman NH (1980). Respiratory consequences of methadone: the response to added resistance to breathing. Am Rev Respir Dis.

[B28] Santiago TV, Pugliese AC, Edelman NH (1977). Control of breathing during methadone addiction. Am J Med.

[B29] Ferrari A, Coccia CP, Bertolini A, Sternieri E (2004). Methadone – metabolism, pharmacokinetics and interactions. Pharmacol Res.

[B30] Dyer KR, Foster DJ, White JM, Somogyi AA, Menelaou A, Bochner F (1999). Steady-state pharmacokinetics and pharmacodynamics in methadone maintenance patients: comparison of those who do and do not experience withdrawal and concentration-effect relationships. Clin Pharmacol Ther.

[B31] Dyer KR, White JM, Foster DJ, Bochner F, Menelaou A, Somogyi AA (2001). The relationship between mood state and plasma methadone concentration in maintenance patients. J Clin Psychopharmacol.

[B32] Kreek MJ, Bart G, Lilly C, LaForge KS, Nielsen DA (2005). Pharmacogenetics and human molecular genetics of opiate and cocaine addictions and their treatments. Pharmacol Rev.

